# The Intracranial Volume Pressure Response in Increased Intracranial Pressure Patients: Clinical Significance of the Volume Pressure Indicator

**DOI:** 10.1371/journal.pone.0164263

**Published:** 2016-10-10

**Authors:** Hung-Yi Lai, Ching-Hsin Lee, Ching-Yi Lee

**Affiliations:** Department of Neurosurgery, Chang Gung Memorial Hospital, Chang Gung University College of Medicine, Taoyuan, Taiwan; Fraunhofer Research Institution of Marine Biotechnology, GERMANY

## Abstract

**Background:**

For patients suffering from primary brain injury, monitoring intracranial pressure alone is not enough to reflect the dynamic intracranial condition. In our previous study, a segment of the pressure-volume curve can be expressed by the parabolic regression model with single indicator “a”. The aim of this study is to evaluate if the indicator “a” can reflect intracranial conditions.

**Methods:**

Patients with traumatic brain injury, spontaneous intracranial hemorrhage, and/or hydrocephalus who had external ventricular drainage from January 2009 to February 2010 were included. The successive volume pressure response values were obtained by successive drainage of cerebral spinal fluid from intracranial pressure 20–25 mm Hg to 10 mm Hg. The relationship between withdrawn cerebral spinal fluid volume and intracranial pressure was analyzed by the parabolic regression model with single parameter “a”.

**Results:**

The overall mean for indicator “a” was 0.422 ± 0.046. The mean of “a” in hydrocephalus was 0.173 ± 0.024 and in severe intracranial mass with slender ventricle, it was 0.663 ± 0.062. The two extreme intracranial conditions had a statistical significant difference (p<0.001).

**Conclusion:**

The indicator “a” of a pressure-volume curve can reflect the dynamic intracranial condition and is comparable in different situations. A significantly larger indicator “a” with increased intracranial pressure is always observed in severe intracranial mass lesions with cerebral edema. A significantly smaller indicator “a” with increased intracranial pressure is observed in hydrocephalus. Brain computed tomography should be performed early if a rapid elevation of indicator “a” is detected, as it can reveal some ongoing intracranial pathology prior to clinical deterioration. Increased intracranial pressure was frequently observed in patients with intracranial pathology. The progression can be differentiated using the pattern of the volume pressure indicator.

## Introduction

Continuous monitoring of intracranial pressure (ICP) is a standard procedure in patients with severe traumatic brain injury. The use of external ventricular drainage (EVD) as an ICP monitor has another important advantage: increased control over ICP through drainage of cerebrospinal fluid (CSF). Increased ICP (IICP) may indicate the worsening of intracranial pathology because of mass lesions or whole brain swelling. However, ICP monitoring alone cannot always predict any structural and functional deterioration caused by progressive growth of contusions, hematomas, brain edema or hydrocephalus. Understanding the intracranial volume-pressure (V-P) relationship can help us determine a reasonable treatment and predict the prognosis of patients with brain damage. The intracranial V-P relationship can be studied using volume-pressure response (VPR) which was defined by Miller [1] as the intracranial pressure change following increases or reductions of ventricular volume. The pressure-volume index (PVI), as defined by Marmarou, is the necessary volume needed to achieve a tenfold increase in the opening pressure.[2, 3] It was considered the key parameter during assessment to determine the compliance of the craniospinal space.[2–6] Elastance is a system parameter that is defined by the pressure change per unit of volume change, which is the corresponding pressure change resulting from any given volume increase in craniospinal contents.[2, 7–10] Intracranial compliance (ICC) is the inverse of elastance and is a measure of the distensibility of the CSF compartment in relation to the intracranial V-P relationship.[2, 7, 10] Although different methods such as VPR, PVI, and ICC have been reported, no reports regarding how to correlate the dynamic intracranial changes have been published.

For patients suffering from a primary brain injury, many secondary injuries compound the initial damage during the following hours and days. Monitoring the intracranial condition and preventing secondary injury is important and indispensable. In our previous study, a segment of the P-V curve delineating abnormal ICP status can be expressed with linear, parabolic and exponential regression models in increased ICP patients. The parabolic regression model is closest to the original ICP curve and is the preferred mathematic model to represent the P-V curve. The regression model is designed with a single indicator “*a*” to reflect the status of the P-V curve.[11] The indicator “*a*” can be compared in different conditions. This study is conducted to evaluate whether the indicator “*a*” can accurately reflect intracranial conditions and be used as a predictive value.

## Materials and Methods

Ethical approval for this study was provided by the Hospital Medical Research Ethics Committee for human research (97-1601B). Written informed consent was obtained from all participants.

The study included patients in the neurosurgical intensive care unit (ICU) with a diagnosis of traumatic brain injury, spontaneous intracerebral hemorrhage (ICH), spontaneous subarachnoid hemorrhage (SAH), and/or hydrocephalus who had external ventricular drainage (EVD) performed whose primary doctor was one of three designated neurosurgeons from January 2009 to January 2010. The initial diagnosis was confirmed by a brain computed tomography (CT) scan. Once informed consent was obtained, the patients were included in the study. Patients were excluded from this study if the EVD function was poor and early tube obstruction occurred within three post-operative days (POD). Patients who stayed in the neurosurgical ICU for less than three days were also excluded. The EVD (“BMI^®^” CSF shunting system) was connected to an ICP monitor and used for drainage of CSF. The ICP was measured by direct ventricular cannulation, and the ICP strain-gauge pressures were connected to the ICP monitor (HP Model 56S; Hewlett Packard, Andover, MA). The ICP values were obtained from the bedside ICU monitor. Once the ICP values reached 20–25 mm Hg, 1 ml of CSF was withdrawn from EVD and the corresponding change in ICP value was recorded. The withdrawn drainage of CSF continued until the final ICP value declined to 10 mm Hg and the whole drainage procedure was completed within one minute. The patients’ Glasgow Coma Scale (GCS) scores, ICP values, and the CSF drainage volume were collected. The postoperative conditions of each patient based on the Glasgow Outcome Scale (GOS) scores were evaluated at discharge and at 6 months thereafter. The Glasgow Outcome Scale is a 5-level score: 1. Dead; 2. Vegetative State; 3. Severe Disability (Able to follow commands/ unable to live independently); 4. Moderate disability (Able to live independently; unable to return to work or school); 5. Good Recovery (able to return to work or school).

The relationship between withdrawn CSF volume and ICP was analyzed with a parabolic regression model to determine the P-V curve as previously documented[11]. The parabolic regression function is defined as “*Y* = *aX*^2^ + 10” to get a single parameter “*a*”, where *Y* is the ICP (mm Hg), and *X* is the CSF volume (mL). The leading coefficient “*a*” in the parabolic regression equation acts as the indicator of the P-V curve. The end point of the CSF drainage process is around an ICP of 10 mmHg. Therefore, the constant in the parabolic regression function is defined as “10”. The different P-V curves are computed by applying parabolic regression equations to each successive VPR value. The sample coefficient of determination (*r*^2^) is calculated as a measure of the closeness-of-fit of the sample regression line to the sample observation. The *F* statistic is used to test the significance of each regression equation.

Another brain CT would be performed if a persistent IICP condition was observed even after CSF drainage, or if neurological deficits or a significant deterioration in GCS were found. Additionally, if a rapid change of indicator “*a*” was detected in IICP patients, a brain CT scan was arranged again to evaluate if any changes in intracranial condition had occurred. The indicator “*a*” was compared at different levels of initial ICP and in different intracranial conditions. The overall brain CT scans in 20 patients were reviewed and analyzed. In the group with hydrocephalus, patients presented with prolonged intracranial hypertension and the last brain CT scans revealed a dilated ventricle with minimal residual primary mass lesion or brain edema. The P-V curves obtained from the condition of hydrocephalus with minimal intracranial mass effect were gathered in Group A. On the other hand, the brain CT sometimes showed severe primary intracranial mass lesion with perifocal edema, brain swelling, and a slender ventricle. The P-V curves obtained from this situation were gathered into Group B. There were still several other intracranial conditions without significant intracranial mass lesion or severe hydrocephalus. There were scattered intracerebral hemorrhage, subarachnoid hemorrhage, mild brain swelling or thin subdural hemorrhage. The P-V curves from other intracranial conditions not including hydrocephalus in Group A or severe intracranial mass lesion in Group B were gathered into Group C. The mean of indictor “*a*” in each group was calculated and Levene’s test was used to assess the equality of variances in different samples. The Kruskal-Wallis one-way analysis of variance (Kruskal- Wallis test) was used to test whether the mean of indicator “*a*” has significant differences for the diverse intracranial conditions in Group A, B, and C. For multiple comparison, the Mann-Whitney U test with Bonferroni correction and Dunn test were used. Differences of p < 0.05 were considered statistically significant. For statistical analysis, we used the software statistical package for the social sciences version 17.0 for windows (SPSS, Chicago, USA) and Microsoft Excel 2007.

## Results

**Fig 1:**
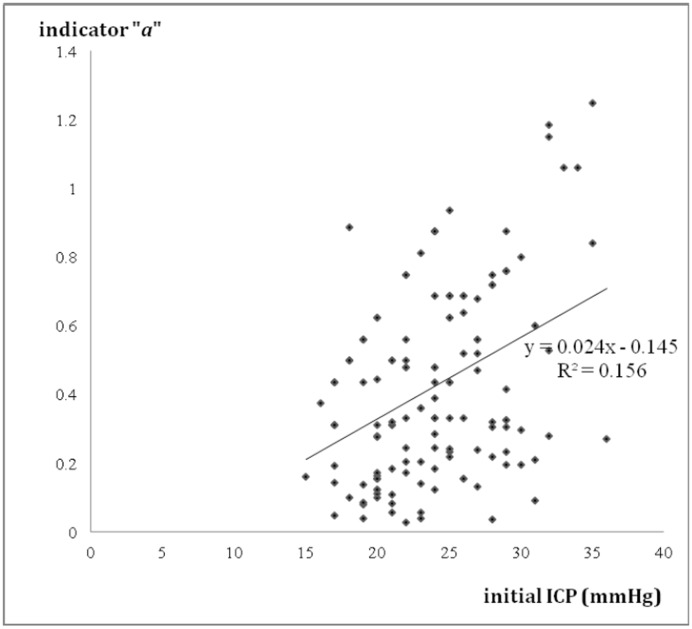
Relationship between initial ICP and the indicator "a" in parabolic equations. N = 139. Regression line is shown; regression equation: y = 0.024x-0.145; R^2^ = 0.156.

There were 20 patients: 14 males and 6 females, with an age range of 50 ± 16 (mean ± SD) years old included in this study. There were seven patients with an initial diagnosis of spontaneous ICH. Nine patients had traumatic brain injury. Four patients had spontaneous SAH with or without hydrocephalus (Table 1). A series of 139 VPR readings were obtained. The different P-V curves were depicted with a parabolic regression equation. All parabolic regression equations had statistical significance (*p* < 0.005) with the *F* statistic (Table 2). The overall mean of the “*a*” indicators in the parabolic equations was 0.422 ± 0.046 (95% confidence interval, CI). The plot of the 139 values of indicator “*a*” in the parabolic regression equations against the initial ICP is shown in Fig 1. The linear regression equation is *y* = 0.024*x*-0.145 (R^2^ = 0.156). Linear regression analysis of indicator “*a*” versus the initial ICP in all P-V curves yielded low coefficients of correlation.

**Table 1:** Demographic details of 20 patient.

Sex	Male	Female
Number of patients	12	8
Age	48±15	53± 11
Diagnosis		
Spontaneous ICH	4	3
Traumatic brain injury	6	3
SAH +/- hydrocephalus	2	2
Admitting GCS		
GCS 3–8	5	3
GCS 9–12	6	4
GCS 13–15	1	1

**Table 2:** An example of F value for the significance of the regression equation.

Model	MSR	MSE	F	CV	Significance
Parabolic	106.67	0.23	456.01	6.61	<0.05

MSR: mean square due to regression = SSR/1;

MSE: mean square due to error = SSE/ (n-2)

SSR: sum of squares due to regression; SSE: sum of squares due to error

F = MSR/MSE

CV: critical value = F_α,1,n-2_(α:significant level = 0.05; degree of freedom:1 and n-2)

There were 40 episodes of CSF drainage in Group A and the mean for indicator “*a*” in this group was 0.173± 0.024 (95% CI) [Table 3]. Another 47 episodes of CSF drainage were in Group B and the mean for indicator “*a*” was 0.663± 0.062 (95% CI). The other 51 episodes of CSF drainage were gathered from Group C and the mean for indicator “*a*” was 0.392 ± 0.071 (95% CI) [Table 3]. The equality of variances between Groups A, B, and C was significantly different using the Levene test (p<0.001). The mean of indicator “*a*” in Group A, B and C compared using the Kruskal- Wallis test showed significant differences between the three groups (*p* < 0.001) (Table 3. The mean of indicator “*a*” in Group A was smallest from our results using the Mann- Whitney U test with Bonferroni correction and Dunn test (*p* < 0.001) [Table 3]. The smaller values of “*a*” were always obtained in patients with prolonged intracranial hypertension and hydrocephalus without significant intracranial mass effect in the brain CT scan. On the other hand, the mean for indicator “*a*” in Group B was significantly higher than the other groups (*p* < 0.001) [Table 3]. In the situation of an intracranial mass lesion with severe perifocal edema, brain swelling, and a slender ventricle, a much higher value of “*a*” was always observed. The mean for indicator “*a*” from other conditions, not including severe intracranial mass effect or hydrocephalus only, in Group C was similar to the mean of overall samples.

**Table 3:** The statistics for the indicator “*a*” in different intracranial conditions.

Group	Initial ICP (mmHg)	Number	Mean “a”(± 95% CI)	Minimum “a”	Maximum “a”
A	17 to 31	40	0.173 ± 0.024^§^	0.027	0.327
B	17 to 35	48	0.663± 0.062^¶^	0.333	1.25
C	15 to 36	51	0.392 ± 0.071	0.04	0.889

Group A: hydrocephalus without significant intracranial mass effect in the brain CT scan.

Group B: severe intracranial mass lesion with perifocal edema, brain swelling and slender ventricle.

Group C: other intracranial conditions, not in Group A or B.

The mean of indicator “*a*” was significantly different between Groups A, B and C with Kruskal- Wallis test (p< 0.001).

^§^ The mean of “*a*” in group A was smallest by Mann-Whitney U test with Bonferroni correction and Dunn test (*p* < 0.001).

^¶^ The mean of “*a*” in group B was highest in these 3 groups (*p* < 0.001).

When the outcome was reviewed after 6 months, there were 6 patients with a GCS of 3–8, 4 patients with a GCS of 9–12 and 8 patients with a GCS of 13–15. From our results, 7 of 20 patients (35%) were observed with a rapid elevation of indicator “*a*” within one day or two successive days after EVD surgery (Table 4). Brain CT scans were performed immediately in these 7 patients when rapidly elevated “*a*” was detected and brain CT scans all revealed significant intracranial pathology. The clinical features and follow-up brain CT findings in the 7 patients were summarized in Table 4. The elevated indicator “*a*” ranged between 0.326 and 0.57 with a mean of 0.473. Four patients (patient 1, 2, 4, and 6) presented with enlarged primary ICH with perifocal edema, brain swelling and slender ventricles during follow-up brain CT scans. They all received medical treatment and CSF drainage because there was no accessible lesion for surgical decompression and they survived brain injury. A delayed contusion hemorrhage that needed surgical decompression was detected in one patient (patient 3) due to a rapid elevation of indicator “*a*” from 0.32 to 0.755 without change in GCS scores. Afterwards, he underwent right side craniotomy for delayed hemorrhage. Two of the 7 patients (patient 5 and 7) died due to the progression of primary brain lesion or new onset intracranial pathology. Patient 5 suffered from a left putaminal hemorrhage and underwent right frontal EVD only. Rapid elevation of the indicator “*a*” was observed on post-operative day (POD) 6 and the follow-up image revealed left ICH progression with brainstem compression. The patient only received conservative treatment and died on POD 15. Patient 7 suffered a left internal carotid artery (ICA) traumatic pseudoaneurysm and diffuse SAH in a traffic accident. Left ICA and right anterior cerebral artery (ACA) territory infarction, severe brain swelling and diffuse vasospasm occurred on POD 4 with the rapid elevation of indicator “*a*” to 0.501 and expired on POD 9. Compared with the GOS after 6 months, the outcome in the 7 patients with rapid elevation was relatively poor amongst the 20 patients. There were 2 patients who died, a GOS of 2 in 3 patients, a GOS of 4 in 1 patient and a GOS of 5 in 1 patient. On the other hand, no patients died among the other 13 patients. The GOS outcomes after 6 months were a GOS of 2 in 3 patients, a GOS of 3 in 2 patients, a GOS of 4 in 3 patients and a GOS of 5 in 5 patients.

**Table 4:** Summary of clinical features in 7 patients with rapid elevation of indicator “*a*”.

Patient no.	Initial diagnosis in brain CT	Motor response of Initial GCS	Elevated “*a*” / duration	Follow-up brain CT findings	Treatment	Outcome
1	Left thalamic hemorrhage, IVH	M4	0.488 / 2 days	Stationary ICH with cerebral edema; brain swelling; slender ventricles	CSF drainage and medical treatment	GOS 2 in 6 months
2	Pontine hemorrhage; IVH; hydrocephalus	M4	0.57 / 2 days	Stationary ICH with perifocal edema; slender ventricles	CSF drainage and medical treatment	GOS 2 in 6 months
3	Left P-T EDH; bilateral fontal and temporal ICH; SAH	M5	0.433 / 1 day	Right frontal and temporal ICH progression; Right F-T-P SDH; brain swelling; slender ventricles	Right F-T-P craniotomy for ICH and SDH removal	GOS 4 in 6 months
4	Bilateral frontal contusion hemorrhage; SAH	M5	0.481 / 2 days	Stationary ICH with perifocal edema; brain swelling; slender ventricles	CSF drainage and medical treatment	GOS 5 in 6 months
5	Left putamial hemorrhage	M4	0.326 / 1 day	ICH progression; perifocal edema; small ventricles	Conservative treatment	death
6	bilateral fontal contusion; left temporal small contusion; SAH	M4	0.51 / 2 days	Stationary ICH with perifocal edema; brain swelling; slender ventricles	CSF drainage and medical treatment	GOS 2 in 6 months
7	Left ICA traumatic pseudoaneurysm; SAH; Hydrocephalus	M5	0.501 / 1 day	Left ICA and right ACA territory ischemia; brain swelling; slender ventricles; diffuse vasospasm	Conservative treatment	death

IVH: intraventricular hemorrhage; ICH: intracerebral hemorrhage; SAH: subarachnoid hemorrhage; EDH: epidural hematoma; SDH: subdural hematoma; CSF: cerebrospinal fluid; M4: withdrawal to painful stimuli in motor response; M5: localized painful stimuli in motor response; F-T-P: fronto-temporo-parietal; ICA: internal carotid artery; ACA: anterior cerebral artery; GOS 2: vegetative status; GOS4: moderate disability; GOS 5: good recovery

### Case Illustrations

#### Case 1

**Fig 2:**
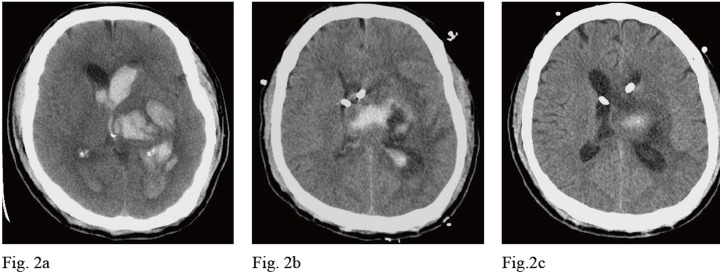
The change of brain CT scans in a spontaneous ICH patient (case 1) in 9 days. Fig 2a shows that the initial brain CT revealed left thalamic hemorrhage, intraventricular hemorrhage, and bilateral dilated ventricle. Fig 2b shows that on POD 6, the brain CT revealed that left thalamic hemorrhage was stationary. The brain parenchyma was tight, especially on the left side. Severe peri-hematoma edema was noted and the brain was shifted to right side. Two EVD tubes were placed in the bilateral frontal horns and the ventricles became slim. Fig 2c shows that the follow-up brain CT on POD 9 revealed that the hematoma and perifocal edema regressed with significantly less mass effect. The brain parenchyma became slack with clear margin of sulci. The EVD tubes were in bilateral enlarged lateral ventricles.

**Fig 3:**
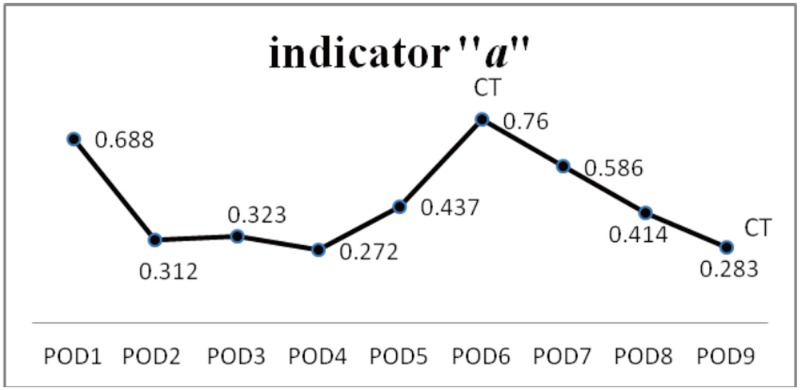
The change of mean of indicator “*a*” in parabolic P-V curve in case 1. The mean of indicator “*a*” in parabolic P-V curve declined from 0.688 on POD 1 to 0.272 on POD 4. However, it started elevating on POD 4 and reached the highest point (0.76) on POD 6. The indicator “*a*” declined again after POD 6 and it was 0.283 on POD 9.

A 48-year-old male patient who had hypertension and hypertrophic cardiomyopathy presented with an acute onset of consciousness change. His GCS was E1V1M4 on arrival in our emergency room (ER). The emergent brain CT revealed left thalamic hemorrhage, intraventricular hemorrhage (IVH) and hydrocephalus [Fig 2a]. Bilateral EVD was performed and he was hospitalized in the ICU post-operatively. Drainage of CSF from EVD was the main method to control intracranial hypertension. The mean of daily changes of the indicator “*a*” is shown in Fig 3. After drainage of CSF to control IICP, the indicator “*a*” with parabolic regression in P-V curve declined from 0.688 on POD 1 to 0.272 on POD 4 [Fig 3]. However, the indicator “*a*” began to increase after POD 4 and reached the highest point (0.76) on POD 6. The patient’s GCS scores remained E1VeM4 during these periods. A brain CT was performed on POD 6 and revealed that the left thalamic hemorrhage was stationary and the ventricle size decreased. The brain parenchyma was tight, especially on the left side, and severe perifocal edema was shown [Fig 2b]. The indicator “*a*” began to decline again after POD 6 and it was 0.283 on POD 9 [Fig 3]. The follow-up brain CT on POD 9 revealed that the left thalamic hemorrhage and perifocal edema regressed with significantly less mass effect and the GCS scores remained the same as day 1. The brain parenchyma became slack and the ventricle size enlarged [Fig 2c]. A VP shunt was performed later. The GOS score was 2 at the postoperative 3-month follow-up.

#### Case 2

**Fig 4:**
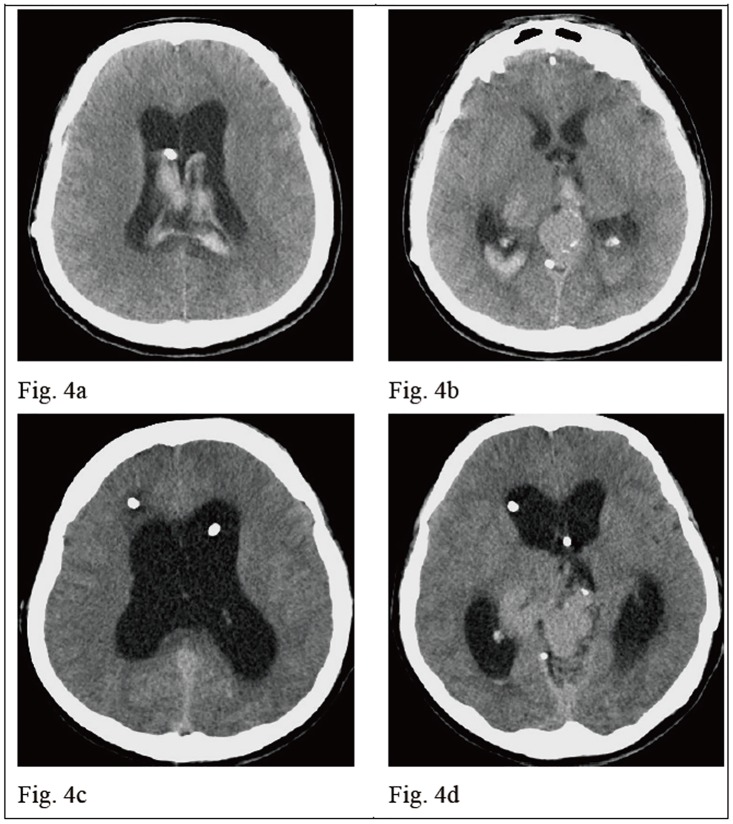
The change of brain CT scans in an intraventricular hemorrhage with hydrocephalus patient (case 2) in 7 days. Fig 4a. and 4b show that the initial brain CT revealed a thalamic vascular lesion and intraventricular hemorrhage in enlarged bilateral ventricles. A right VP shunt tube was placed in the right frontal horn. Fig 4c. and 4d show that the follow-up brain CT on POD 7 revealed a stationary thalamic vascular lesion and regression of intraventricular hemorrhage. Hydrocephalus was persistent and two EVD tubes were placed in the bilateral frontal horns.

**Fig 5:**
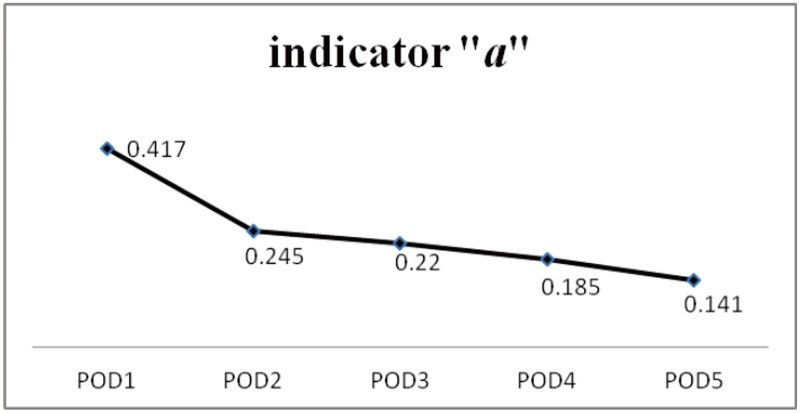
The change of mean of indicator “*a*” in parabolic P-V curve in case 2. The mean of parameter “*a*” declined gradually from 0.417 on POD 1 to 0.141 on POD 5.

**Fig 6:**
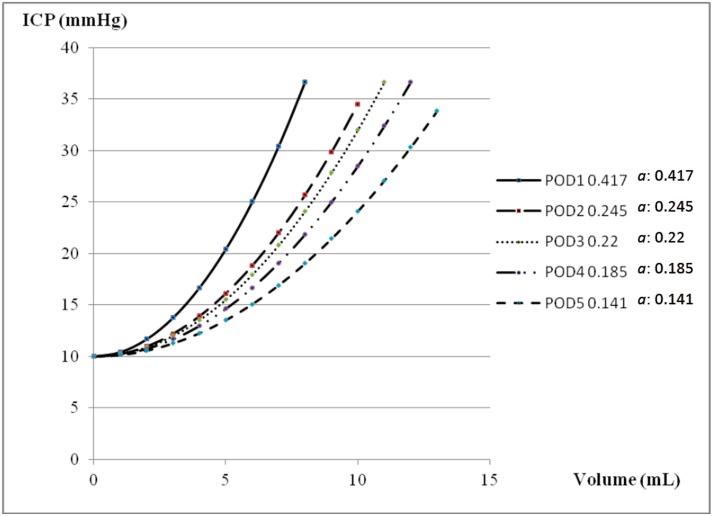
Example of different P-V curves in parabolic regression equations with different indicator “*a*” in one patient from case illustration 2. The steepest P-V curve was derived from POD 1 with an indicator “*a*” of 0.417. From left to right, the P-V curve became more and more flat and the indicator “*a*” in each P-V curves declined from POD 1 to POD 5.

A 31-year-old male patient had history of thalamic and brainstem arteriovenous malformation and he underwent stereotactic radiosurgery and right VP shunt 4 years ago. He presented with a progressive headache for 4 days. He was drowsy and experienced general malaise with left side limb weakness noted when he arrived in our emergency room. The brain CT revealed IVH and bilateral dilated ventricles [Fig 4a and 4b]. The right VP shunt function became poor and was removed. Bilateral EVD was performed and he was hospitalized in the ICU post-operatively. Drainage of CSF from the EVD was used to treat the IVH and hydrocephalus and control intracranial hypertension. His consciousness regained clarity and alertness on POD 1. The mean of indicator “*a*” declined gradually from 0.417 on POD 1 to 0.141 on POD 5 [Fig 5] and he was transferred to an ordinary ward. The follow-up brain CT in POD 7 revealed regression of IVH but hydrocephalus was still existent [Fig 4c and 4d], therefore a VP shunt was performed. The P-V curve in this patient is different with a changeable indicator “*a*”. The progression of the P-V curves from POD1 to POD5 in case 2 is shown in Fig 6.

#### Case 3

**Fig 7:**
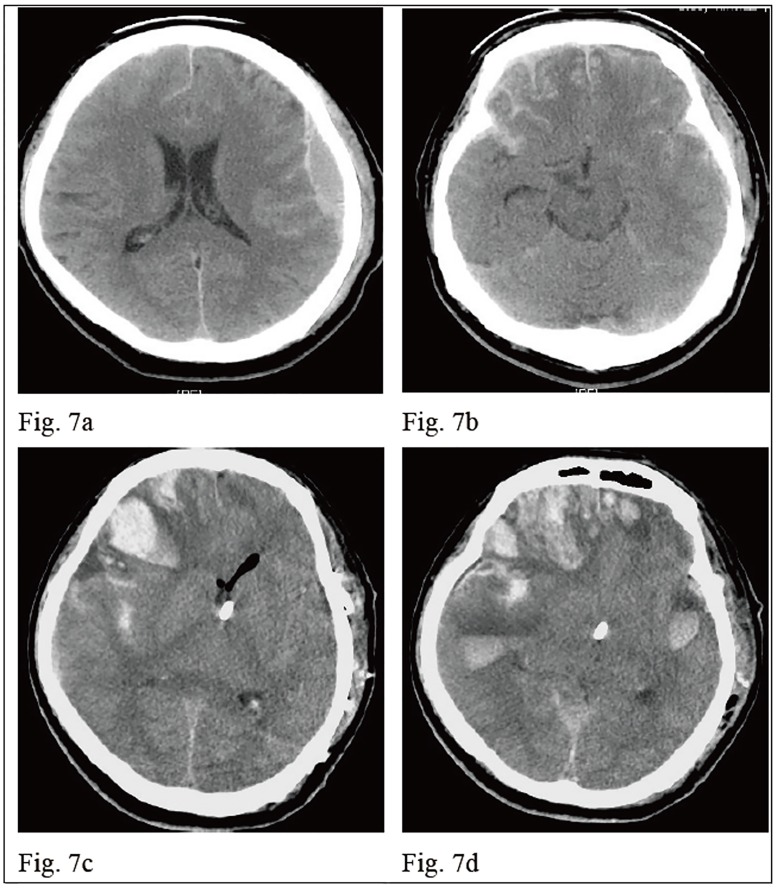
The progression of brain CT scans in a traumatic brain injury patient (case 3). Fig 7a. and 7b show that the initial brain CT revealed left fronto-temporal EDH and bilateral frontal and temporal small contusion hemorrhages, and SAH. Fig 7c. and 7d show that the follow-up brain CT after left craniotomy on POD 1 revealed right frontal and temporal contusion hemorrhage progression and right SDH with a midline shift to the left, left frontal and temporal contusion hemorrhages, pneumocephalus, diffuse brain swelling and bilateral slender ventricles. The EVD tube was placed in the left frontal horn.

**Fig 8:**
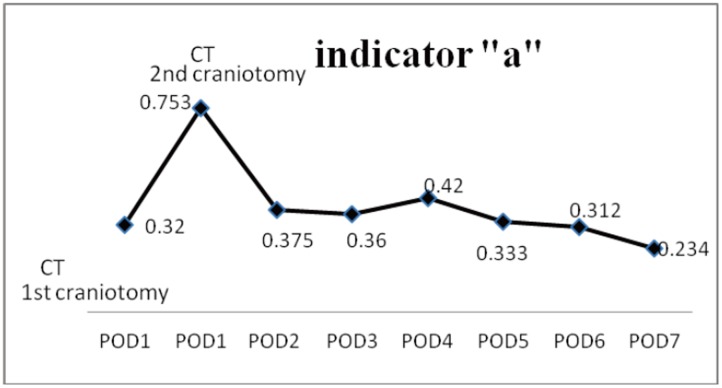
The change of mean of indicator “*a*” in parabolic P-V curve in case 3. The indicator “*a*” elevated rapidly from 0.32 to 0.753 during POD 1. After a second craniotomy, the indicator “*a*” declined to 0.375. The mean of indicator “*a*” from POD 2 to POD 7 fluctuated between 0.234 and 0.42 and no more rapid elevation of “*a*” was noted after the second craniotomy.

A 65-year-old male patient suffered from a head injury during a traffic accident. He was found with a change in consciousness immediately after the injury and his GCS was E1V1M5 on arrival in our ER. The initial brain CT revealed left fronto-temporal (F-T) epidural hematoma (EDH) and bilateral fontal and temporal small contusion hemorrhage, and SAH [Fig 7a and 7b]. He underwent left side craniotomy for EDH removal and left frontal EVD for ICP monitoring. After craniotomy, he was admitted to the ICU. However, a dramatically elevated indicator “*a*”, from 0.32 to 0.755, was noted on POD 1 without changes in GCS scores [Fig 8]. The follow-up brain CT revealed right frontal and temporal contusion hemorrhage progression and right subdural hematoma (SDH) with a midline shift to the left, pneumocephalus, and bilateral slender ventricles [Fig 7c and 7d]. He underwent urgent right side craniotomy for ICH and SDH removal. After the surgery, the indicator “*a*” declined to 0.375 and it fluctuated between 0.234 and 0.42 during the first week [Fig 8]. He had a smooth recovery course after the second craniotomy and he recovered, regaining clear consciousness eventually. He underwent a VP shunt on POD 21. His GOS score at the postoperative 3-month follow-up was 4.

## Discussion

ICP monitoring is a common neurosurgical practice for patients with intracranial pathology. ICP values are used as a measure of pathology, as an indicator for treatment response, and to monitor cerebral perfusion. The upper limit of normal ICP in adults is usually considered to be 15 mm Hg, although the usual range is 5 to 10 mm Hg. ICP can be measured using intraventricular, intraparenchymal, subdural, or epidural devices. Ventriculostomy remains the gold standard for monitoring ICP because of its accuracy and ease of calibration. Access to CSF for dynamic testing and drainage to control ICP are additional benefits. There is no uniform agreement about the critical level of ICP beyond which treatment is mandatory.[7] Saul and Ducker demonstrated benefits in treating patients whose ICP was greater than 15 mm Hg, as compared with a group of patients treated for ICP greater than 25 mm Hg.[12] According to the guidelines for the management of severe traumatic brain injury in 2007, 20 to 25 mm Hg is an upper threshold above which treatment to lower ICP should generally be initiated.[13] In this study, the treatment threshold was 20 to 25 mm Hg in patients with intracranial hypertension. Once the ICP values reached 20–25 mm Hg on the bedside monitor, drainage of CSF was performed until the ICP value declined to 10 mm Hg. But occasionally initial ICP values were beyond 25 mm Hg or less than 20 mm Hg at the beginning of the CSF drainage procedure.

Comprehending the intracranial V-P relationship in patients with increased ICP can help us decide on a treatment plan and predict the prognosis. Miller and Garibi documented the concept of volume pressure response (VPR). A volumetric change in patients of 1 ml CSF in one second was used and the resultant immediate change in ICP was termed the VPR. It is expressed in mm Hg/ml and is used as an index of intracranial elastance.[1, 8] The VPR was considered to be related to the amount of brain shift seen in patients with head injuries and brain tumors, and is reduced following surgical decompression.[8] In Miller’s report, the VPR value was used as an indicator in patients with temporal lobe contusions of moderate degree in whom the surgeon is uncertain whether to use medical or surgical decompression. If the VPR is greater than 3 mm Hg/ml even if the ICP is not significantly elevated at the time, the patient requires decompression.[8] Miller[8] and Tans[6] considered a VPR of 2 mm Hg/ml or less to be normal and a VPR greater than 4 mm Hg/ml to be definitely pathological. However, it is questionable that a single measurement of VPR can represent an entire P-V curve. A single VPR provides only a small part of the intracranial P-V curve with a 1ml width of CSF volume. The VPR also has highly variable dynamic changes and a single VPR cannot display the intracranial V-P relationship entirely. Another consideration is that fluid injection into the intracranial space to get a VPR must be prohibited when there is an increased ICP status. Any additional volume placed into the intracranial space may cause further structural and physiological damage to the brain.

Marmarou documented that the plotting of ICP logarithmically against volume produces a straight line. Its slope is the pressure-volume index (PVI), or the calculated volume in milliliters needed to raise ICP by a factor of 10. PVI is considered a parameter that expresses intracranial compliance.[2, 3, 7] It is considered that a PVI of 25 to 30 ml is normal in adults.[7, 14] Shapiro and Marmarou reported that a reduced PVI was an accurate predictor of impending intracranial hypertension.[14] When intracranial compliance is reduced by a pathologic process, PVI diminishes and small volume changes result in much greater pressure changes. A PVI less than 10 to 13 ml was considered a pathological intracranial compliance and the critical PVI threshold for brain-injured patients.[6, 7] In fact, a PVI of 10 to 13ml may occur at different areas of a baseline ICP, either on the near horizontal part of the P-V curve, or very close to the vertical part. The two conditions need obviously different treatment strategies. The PVI value is directly calculated from a single point of VPR. As with VPR, the PVI cannot reflect the intracranial P-V relationship entirely. The PVI refers only to the pressure changes caused by uncompensated rapid volume changes. However, pathological volume changes of intracranial contents do not occur rapidly, and the PVI provides little information about a patient’s remaining compensatory abilities.[6, 9, 13] Tans, *et al*. concluded that the VPR does not provide reliable information about the V-P relationship because the correlation coefficient calculated from regression analysis of VPR and PVI was as low as -0.33.[6] The PVI magnitude depends on many other factors such as neural axis volume, CSF outflow resistance, systemic arterial pressure, and arterial pCO2. [6] There is possible sampling error when obtaining a single PVI based on different times and baselines of ICP. Fluid injection into the intracranial space to get a PVI should also be avoided in patients with increased ICP.

A single intracranial compliance (ICC) of a patient at one time cannot reflect the dynamic intracranial conditions. Kiening, *et al*., documented the clinical practice of continuous monitoring of intracranial compliance for patients with closed severe traumatic brain injury.[10] Pathological episodes for ICP (ICP>20 mm Hg > 10 min) and continuous ICC (cICC < 0.5ml/mm Hg > 10 min) were defined. From a total of 225 episodes of increased ICP, only 37 were detected by a preceding low cICC. In contrast, prolonged pathological cICC was present in 118 episodes, which were not associated with high ICP values. It also revealed broad scattered correlation coefficients, ranging from 0.05 to 0.52 between cICC and ICP. The low detection rate of an impending ICP rise of only 16% substantiates the low impact of valid cICC measurements on episodes of markedly elevated ICP. Use of single ICC is limited as it is data calculated from the P-V curve and represents a single value at a particular aspect of intracranial condition. Multiple values are needed to see the intracranial dynamic changes. In addition, the cICC monitoring in clinical practice requires a time delay due to cICC assessment which needs 200-data points to be integrated.[10]

Although VPR, PVI, and ICC were documented to increase understanding of the intracranial V-P relationship, no reports can explain how to correlate them with the dynamic intracranial conditions immediately. VPR, PVI, and ICC are not as widely applied in current clinical practice as ICP monitors are nowadays. The V-P relationships can be depicted by graphing the successive response of ICP to volume added or withdrawn from the ventricle.[7, 8, 14, 15] In our previous study, the parabolic regression equation is a preferable mathematic model to acquire a segment of the P-V curve in patients with an increased ICP status. The P-V curve can be depicted precisely by parabolic regression especially when the ICP is within the range of 10 to 40 mmHg.[11] The segment of P-V curves with an ICP between 10–40 mmHg is always observed in patients with brain injury who need aggressive monitoring and treatment. The regression model is designed to have a single parameter “*a*” as an indicator, and it can display the dynamic changes in intracranial V-P relationship and is comparable in different conditions. Most importantly, Miller has documented that the P-V curves change in patients based on different conditions. In his report, mannitol and steroids reduce the VPR much more than they reduce ICP itself. It was suggested that these agents alter the shape of the P-V curve, flattening it and creating a situation in which the brain is much more tolerant to the addition of volume.[8] Different patients will have different shapes of P-V curves. Moreover, in any one patient, the P-V curve is different with a changeable indicator “*a*” representing diverse intracranial conditions. The change of indicator “*a*” indicates a change in the P-V curve and may represent the deterioration or regression of an intracranial lesion. A series of P-V curves can provide an easier way to realize the intracranial V-P relationship and the course of brain injury. In one patient with a status of increased ICP, as the indicator “*a*” in the parabolic P-V curve decreased, the P-V curve became more flat [Fig 6]. In clinical practice, a smaller indicator “*a*” in the P-V curve represents better compliance of the brain with better spatial compensation ability.

No direct relationship can be defined between the level of ICP and the neurological status of patients at that time or the subsequent clinical course. Increased ICP level does not provide reliable correlation to the indicator “*a*” because the linear regression analysis of indicator “*a*” versus the initial ICP yielded low coefficients of correlation (R^2^ = 0.156) [Fig 1]. From our results, some higher values of indicator “*a*” were obtained from patients with less elevated ICP status with an ICP < 20 mm Hg. Even patients with greater IICP status (ICP > 25 mmHg) could yield a smaller indicator “*a*” that was below the linear regression line [Fig 1]. In daily clinical practice, IICP does not always represent that intracranial compliance is unfavorable. The intracranial spatial compensation ability may be still acceptable despite IICP status. However, a higher value of indicator “*a*” may be a clue or sign of poor intracranial spatial compensation ability. From our results, the patients in Group B significantly had the highest value of indicator “*a*” when compared to the other conditions (*p* < 0.001). In patients with IICP that have a similar clinical conditions and the same GCS scores, a larger indicator “*a*” is usually detected in patients with severe intracranial parenchymal pathology and mass effect such as case illustration 1 [Figs 2a and 2b and 3]. A smaller indicator “*a*” is usually observed in patients with relatively benign intracranial conditions. The mean of indicator “*a*” in Group A was smallest with statistical significance (*p* < 0.001). If patients showed prolonged IICP status with a need for persistent CSF drainage and a smaller indicator “*a*”, the brain CT usually revealed hydrocephalus alone such as case illustration 2 [Figs 4c and 4d and 5]. The brain CT of case illustration 1 on POD 9 also revealed the regression of ICH and cerebral edema but persistent bilateral dilated ventricles [Fig 2c] with a smaller indicator “*a*” [Fig 3]. Throughout the different intracranial conditions between Group A, B and C, patients may present with similar intracranial hypertension that requires CSF drainage from EVD. The mean of indicator “*a*” reveals significant difference between the two extreme intracranial conditions in Group A and B [Table 2]. If a persistent smaller indicator “*a*” was noted in patients with IICP, hydrocephalus should be considered and a VP shunt is usually indicated. In this study, 12 patients (60%) had prolonged intracranial hypertension and hydrocephalus and they all underwent VP shunt within one month after primary brain injury. If a higher indicator “*a*” was observed, intracranial mass lesion with brain swelling was usually the main cause of intracranial hypertension. Surgical decompression should be considered according to the brain CT findings.

For patients suffering from primary brain injury, secondary injuries can worsen the clinical conditions in the following hours and days. Monitoring the intracranial conditions and ongoing secondary brain injury is necessary. A series of dynamic indicator “*a*” in one patient can reflect the dynamic changes of intracranial V-P relationship and corresponding intracranial pathology, allowing ongoing intracranial deterioration to be detected earlier in IICP patients with the same GCS scores. In case illustration 1, the highest indicator “*a*” was detected on POD 6 with the same GCS scores as POD 1. However, the brain CT at that time revealed progression of ICH and cerebral edema with significant mass effect [Figs 2b and 3]. From our results, 7 of 20 patients (35%) were observed with a profound elevation of indicator “*a*” during one or two successive days after EVD surgery [Table 2]. All brain CT scans in these 7 patients revealed significant intracranial deterioration which explained the decreased intracranial compliance. The follow-up brain CT scans sometimes revealed significant primary ICH progression like that found in patients 3 and 5 who needed surgical decompression. Illustrated case 3 underwent contralateral craniotomy for surgical decompression before any indication of the clinical deterioration in neurological status and GCS scores was seen. Although 4 of 7 patients (Table 2, patients 1, 2, 4 and 6) with rapidly elevated “*a*” presented no obvious enlargement in primary ICH, the progression of peri-hematoma edema and whole brain swelling with slender ventricles was observed. Patient 8 developed a new intracranial pathology with left ICA and right ACA territory infarction along with a rapidly elevated indicator “*a*”. The indicator “*a*” in two mortality cases (Table 2, patients 5 and 7), increased progressively and EVD was obstructed and the intracranial spatial compensation ability was lost. Therefore, a rapid elevation of the indicator “*a*” usually indicates ongoing intracranial deterioration although the patient may have similar IICP status and the same GCS scores after primary brain injury. A rapid change of indicator “*a*” may precede the clinical deteriorations. A brain CT scan should be arranged earlier to evaluate if any surgical decompression should be performed before the deterioration in clinical conditions and GCS scores is seen. The elevation of indicator “*a*” in these 7 patients ranged between 0.326 and 0.57 with a mean of 0.473.

Take a glance at our results; the data for indicator “*a*” was all gained from a situation of IICP. Because the CSF was drained when the ICP reached 20-25mmHg, it was difficult to compare different patients using the primary ICP data. The pattern of IICP and intracranial condition can be differentiated using the indicator “*a*”. In this study, the indicator “*a*” is used as a relative value. Comparison of the indicator “*a*” of the P-V curve at different conditions in one patient is suitable, but not for comparison among patients. A series of P-V curves can offer information about the changes in intracranial conditions and the course of brain injury. The rapid change of the indicator was usually observed within 3–7 days of primary brain injury. It revealed that a rapid deterioration of intracranial condition and secondary brain injury usually happened at the acute stage. The trend for the change of indicator “*a*” all declined gradually in patients who became stable after treatment. The indicator “*a*” reached its relative lowest point in patients before they were transferred to a general ward (Figs 3, 5 and 8). The absolute value of the indicator “*a*” that represents a favorable or unfavorable intracranial compliance needs further investigation. Furthermore, with the dynamic changes of indicator “*a*”, the intracranial consequence of all therapeutic modalities (hyperventilation, fluid resuscitation, mannitol, anesthesia, chest physiotherapy, and barbiturate therapy, etc.) can also be assessed and adjusted to protect against secondary brain injury.

## Conclusion

The parabolic regression model is a reliable mathematic model to acquire a segment of the P-V curve in patients with increased ICP. The single indicator “*a*” can reflect the status of the P-V curve and the dynamic change of the intracranial V-P relationship and is comparable at different times. A series of P-V curves can offer information about the changes in intracranial conditions and the course of brain injury. The increased ICP level does not provide reliable correlation with the indicator “*a*”. In clinical practice, a smaller indicator “*a*” of the P-V curve represents better compliance of the brain with better spatial compensation ability. A significantly higher indicator “*a*” is always observed in patients with severe intracranial mass lesion with cerebral edema and slender ventricles. On the other hand, a significantly smaller indicator “*a*” is always observed in patients with relatively benign intracranial lesions such as hydrocephalus alone. Even in patients presenting similar intracranial hypertension and same GCS scores, a rapid elevation of the indicator “*a*” is usually observed along with intracranial condition deterioration. Brain CT scans should be performed early if a rapid elevation of indicator “*a*” is detected in IICP patients even with the same GCS scores as it can reveal some ongoing intracranial pathology before the visible deterioration of clinical conditions. Some surgical decompression procedures can also be performed early if the indicator “*a*” indicates the need. An absolute value of indicator “*a*” that represents a good or bad intracranial compliance needs to be further investigated. The volume pressure indicator “*a*” could offer a more effective, meaningful, and safe method in clinical observation for neurosurgical critical care. A larger study with more patients and different intracranial conditions needs to be performed in the future to strengthen the value of the volume pressure indicator “*a*” for clinical application.
